# Mitigation of microbial biocontamination in cyanobacterial ethanol synthesis via alginate encapsulation

**DOI:** 10.1186/s40643-026-01076-7

**Published:** 2026-05-26

**Authors:** Xiangxiao Liu, Xiang Liu, Yanfei Pan, Huili Sun, Bo Liang, Jiahui Sun, Tianzhong Liu, Guodong Luan, Xuefeng Lu

**Affiliations:** 1 Energy-Rich Compounds Production By Photosynthetic Carbon Fixation Research Center, Shandong Key Lab of Applied Mycology, College of Life Sciences, Qingdao Agricultural University, Qingdao, 266109 China; 2 State Key Laboratory of Photoelectric Conversion and Utilization of Solar Energy Qingdao, Institute of Bioenergy and Bioprocess Technology, Chinese Academy of Sciences, Qingdao, 266101 China; 3 Shandong Energy Institute, Qingdao, 266101 China; 4 Qingdao New Energy Shandong Laboratory, Qingdao, 266101 China

**Keywords:** Cyanobacteria, Ethanol, Biocontamination, Alginate encapsulation, Microbial contaminants

## Abstract

**Background:**

Sustainable biofuels have spurred interest in cyanobacterial ethanol production, yet large-scale application is severely hindered by microbial contamination—a devastating challenge that lacks universally effective, biocompatible mitigation strategies. Traditional methods such as pH manipulation or antibiotic application are often physiologically incompatible, environmentally unsustainable, or ineffective against diverse contaminant consortia.

**Results:**

Here, we propose and validate alginate encapsulation as a physical barrier strategy to address this challenge. Alginate, a biodegradable polysaccharide derived from brown algae, forms a porous hydrogel matrix that encapsulates cyanobacterial cells. This matrix blocks direct contact, uptake or ingestion by microbial contaminants while allowing the efficient diffusion of gases (CO_2_, O_2_), light, and nutrients to maintain photosynthetic and metabolic functions. Using our previously engineered, high-performance ethanol-producing strain (EP), we find that unprotected cultures rapidly collapse and cease ethanol production upon inoculation with a contaminant consortium, with cumulative ethanol yield falling to undetectable levels, whereas encapsulated cells sustain normal growth and photosynthetic activity under identical contamination pressure. After rinsing and re-cultivation, the encapsulated biomass partially restored ethanol productivity, achieving a cumulative titer of 320 mg/L over 4 days post-recovery. This suggests that the protective strategy is non-invasive and enables functional recovery of the production system following contamination exposure.

**Conclusions:**

This study demonstrates that alginate encapsulation represents a promising strategy to mitigate microbial contamination, with the potential to enhance the technical resilience and operational stability of cyanobacterial biofuel production.

**Graphic abstract:**

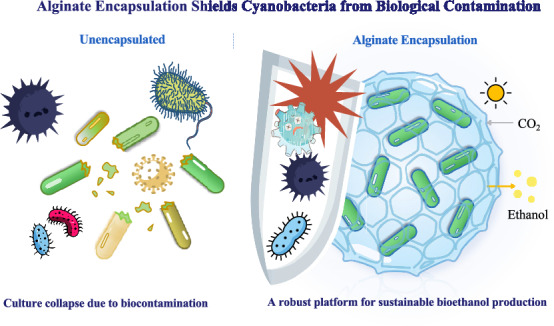

**Supplementary Information:**

The online version contains supplementary material available at 10.1186/s40643-026-01076-7.

## Introduction

The global urgency to mitigate greenhouse gas emissions and address fossil fuel depletion has spurred intensive research into sustainable biofuel production (Singh et al. 2025; El-Araby 2024). Cyanobacteria, as photoautotrophic prokaryotes, offer a transformative platform for renewable ethanol synthesis by directly converting atmospheric CO_2_ and solar energy into biofuels—eliminating the “food-versus-fuel” conflict of first-generation bioethanol and reducing the high pretreatment costs of lignocellulosic feedstocks (Yadav et al. 2021; Mahmood et al. 2023; Andrews et al. 2021; Sabat et al. 2025; Liu et al. 2025). Through metabolic engineering, researchers have successfully constructed cyanobacterial cell factories by introducing heterologous pyruvate decarboxylase (*pdc*) and alcohol dehydrogenase (*adh*) genes. This strategy has achieved notable ethanol titers and productivities in laboratory-scale studies (Dexter and Fu 2009; Wang et al. 2020; Gao et al. 2012). However, translating these lab-scale successes to large-scale outdoor cultivation remains severely hindered by unresolved biocontamination issues (Dexter et al. 2015; Day et al. 2017).

Fast-growing and diverse microbial communities (including bacteria, protozoa, and other microorganisms) represent a critical bottleneck for the industrial application of cyanobacterial cultures (Novoveská et al. 2023; Karuppasamy et al. 2018; Molina-Grima et al. 2022). These contaminants are ubiquitous in aquatic environments and outdoor photobioreactors, where they compete with cyanobacteria for nutrients, secrete harmful metabolites, or directly prey on them (Shi et al. 2021; Weger et al. 2024). This leads to rapid depletion of the cyanobacterial cells, disruption of the carbon fixation-ethanol synthesis cascade, and ultimately complete failure of the production process (Toda et al. 2024). Previous efforts to control contamination have encompassed both broad and targeted strategies. On the broad front, conventional approaches such as pH elevation (Zhu et al. 2017; Touloupakis, et al. 2016), selective nutrient utilization (Toda et al. 2021; Selão et al. 2019) or antibiotic addition (Xu et al. 2021), have been widely explored. Meanwhile, more precise genetic and metabolic engineering strategies have also been investigated, including modifying surface structures like O-antigens to deter specific herbivores (Simkovsky et al. 2012), or altering cell surface charge and hydrophobicity to reduce biofilm formation and predator adhesion (Li, et al. 2021). However, a fundamental constraint in deploying such targeted interventions in open, large-scale cultivation is the unpredictable and heterogeneous nature of environmental contaminants. For instance, the high-pH strategy, which aims to inhibit specific product-consuming competitors (e.g., *Pantoea ananatis*), is inherently limited because its efficacy depends on the unique physiological vulnerability of a single contaminant type (Zhu et al. 2017). Moreover, environmental regulation strategies such as maintaining high nitrogen or phosphorus concentrations often impair cyanobacterial growth, pose environmental risks, or show limited efficacy against complex and adaptive contaminant communities (McBride et al. 2014; Li et al. 2021; Shi et al. 2024; Ernst et al. 2005). Even genetic engineering strategies, such as O-antigen modification or biofilm intervention, are frequently designed to target specific protozoa like amoebae and therefore lack broad applicability against complex contaminant consortia.

As a physical barrier, microencapsulation using natural polymers has been widely employed in microbial biotechnology. However, its application to mitigate microbial contamination in cyanobacterial biofuel production remains underexplored (Chen et al. 2021; John et al. 2011; Jíménez-Arias et al. 2022). Alginate, a biocompatible and biodegradable polysaccharide derived from brown algae, possesses unique advantages for photosynthetic organisms: its hydrated porous hydrogel network allows efficient diffusion of CO_2_, light, and metabolic products (e.g., ethanol) while has the potential to act as a size-exclusion barrier against larger predators such as bacteria and protozoa (Leino et al. 2012; Wawszczak et al. 2024; Abbasi et al. 2024). Unlike chemical or stress-based strategies that often impair cyanobacterial physiology, alginate encapsulation provides a non-invasive shield, protecting cells without interfering with their intrinsic growth and metabolic activity (Kong et al. 2003; Bustamante et al. 2025; Kosourov and Seibert 2009; Covarrubias et al. 2012; Bustos-Terrones 2024).

In this study, we investigated whether alginate encapsulation could protect ethanol-producing cyanobacterial cell factories from complex microbial contamination. Using our previously engineered, high-performance ethanol-producing strain *Synechococcus* sp. PCC 7002 derivative (Wang et al. 2020), we show that alginate encapsulation creates a physical barrier that effectively shields the cyanobacterial cells while preserving their photosynthetic activity and ethanol synthesis capacity. These findings provide new insights into biocontamination management and offer a potential strategy to enhance the viability and ethanol production resilience of photosynthetic cell factories against diverse microbial predators and competitors, and may support further development to address scale-up barriers for cyanobacteria-based biofuel systems.

## Materials and methods

### Cyanobacterial strain and pre-cultivation conditions

*Synechococcus* sp. PCC 7002 derivative 1 + 2PA (hereafter designated EP) was used in this study (Wang et al. 2020). The ethanol biosynthesis pathway genes (*pdc* and *adh*) were genomically integrated into the chromosome of *Synechococcus* sp. PCC 7002 to construct this strain, ensuring genetic stability throughout the experiment. The cultivation conditions and operational procedures for strain EP were strictly consistent with the protocols described in the aforementioned literature (Wang et al. 2020). Pre-cultures were grown in sterile 1 × A + medium in 50 mL flasks at 30 °C with continuous shaking (150 rpm) under white light illumination at 50–70 μmol photons/m^2^/s.

### Establishment and maintenance of a complex microbial contaminant consortium

A complex microbial consortium was isolated from failed, contaminated outdoor pilot-scale cultures of strain EP. Microscopic examination (phase-contrast and inverted light microscopy, 400 × magnification) suggested the presence of diverse contaminating microorganisms, including bacteria, flagellates, protozoa and other motile microorganisms of various morphologies, all of which were documented photographically (see Results). This contaminant consortium was maintained by weekly sub-culturing in mixed culture with EP as the primary nutrient source. Specifically, a fresh EP suspension (OD_730_ = 1.0) was added to the contaminant culture at a volume ratio of 1:5 (EP suspension: contaminant culture) every 7 days. Only consortium batches with stable microbial density (determined by microscopic observation) and consistent morphological diversity were used for subsequent contamination challenge experiments, ensuring reproducible contamination pressure across all experimental treatments.

### Encapsulation of ethanol-producing cyanobacterium EP in sodium alginate

For the encapsulated group, 2% (w/v) sodium alginate solution and 2% (w/v) calcium chloride (CaCl_2_) solution were first prepared and sterilized by autoclaving. When the ethanol-producing cyanobacterium EP in the pre-culture reached the mid‑exponential growth phase, an appropriate volume of the cyanobacterial suspension was gently mixed with an equal volume of sterile 2% (w/v) sodium alginate solution to ensure the total cyanobacterial cell count was consistent with that of the control group. Beads were prepared by extruding a homogeneous cyanobacterial-alginate mixture through a 1 mL syringe fitted with a 0.6 × 25 mm needle into a sterile 2% (w/v) CaCl_2_ cross-linking solution. After solidification, the formed alginate beads encapsulating EP cells were collected by filtration, rinsed several times with sterile A + medium to remove residual CaCl_2_, and then inoculated into an appropriate volume of sterile A + medium for subsequent cultivation. For the control group, an equal volume of EP cyanobacterial suspension (with the same cell count as the encapsulated group) was directly inoculated into sterile A + medium without encapsulation treatment. Each condition included at least three biological replicates.

### Cyanobacterial cultivation conditions and ethanol collection

For the unencapsulated control (standard column cultivation), EP cells were resuspended in 50 mL of fresh 2 × A + medium (twice the concentration of standard A + medium) to an initial optical density at 730 nm (OD_730_) of approximately 0.1. For the alginate microbead-encapsulated treatment, 5 mL of the cyanobacteria-alginate microdroplet suspension was transferred to the column, with 45 mL of fresh 2 × A + medium supplemented to a final culture volume of 50 mL (ensuring consistent initial cell count with the unencapsulated control). Unless otherwise mentioned, the cells were cultivated at 30 °C under continuous white-light illumination at 200 μmol photons/m^2^/s. The incident photosynthetically active radiation was measured using a LI-250A light meter (LI-COR) with a quantum sensor positioned at the center of the vessel wall. For encapsulated cultures, due to the turbidity of the beads, light attenuation within the vessel was not directly measured, nor were intra-bead light gradients assessed. The culture was aerated with a sterile mixture of air and 3% (v/v) CO_2_ (filtered through 0.22 μm membrane), which served as the carbon source. Ethanol produced during cultivation was collected using an ice-cooled condenser connected to the culture column outlet. Specifically, 25 mL of deionized water was pre-added into the collection tube connected to the condenser, and ice bath was consistently maintained throughout the collection process to ensure efficient condensation. On each sampling day, the entire volume of condensate in the collection tube was transferred to a sterile container. The tube was then replenished with 25 mL of fresh deionized water for continued collection.

### Cleaning and surface decontamination of alginate microbeads

To remove contaminants adhering to the surface of alginate microbeads without compromising the viability of the encapsulated cyanobacteria, the following cleaning protocol was adopted, prioritizing gentle physical removal of contaminants and avoiding harsh chemical treatments: The bead suspension was poured onto a sterile filter cloth to drain the liquid phase. The microbeads were subsequently rinsed three times with sterile water to thoroughly eliminate turbid surface liquid containing contaminants. The beads were collected, gently mixed, and then immersed in A + medium containing chloramphenicol (10 μg/mL) and spectinomycin (50 μg/mL). These antibiotics were chosen based on the inherent antibiotic resistance of the engineered EP strain, and the suspension was incubated with shaking at 100 rpm for 30 min. Visible impurities were then manually picked out from the suspension with a sterile pipette tip, after which the microbeads were subjected to an additional rinse step. Finally, the treated microbeads were directly transferred into the culture column, with all aforementioned operations conducted under strict aseptic conditions.

### Ethanol concentration determination

Ethanol concentration was quantified for both the condensed sample (collected via the condenser) and the culture medium. For each sample type, 1 mL of liquid was processed as follows. First, the sample was centrifuged at 12,000 × g for 10 min to pellet any cells or debris. The resulting supernatant was then carefully transferred to a clean 1.5 mL microcentrifuge tube. The supernatant was diluted with deionized water as necessary to ensure the ethanol concentration fell within the linear detection range of the assay kit. Finally, the ethanol concentration in the diluted sample was determined using a commercial Ethanol Assay Kit (Beyotime, S0240M). Total cumulative ethanol yield was calculated as the sum of ethanol recovered from the condensate (normalized to culture volume) and that remaining in the broth.

### Assessment of cell viability and photosynthetic activity in encapsulated cells

To quantitatively assess cell viability and photosynthetic performance in encapsulated samples, alginate beads were first dissolved using sodium citrate as described previously (Singh and Ducat 2022). Beads were collected by sedimentation and incubated in 100 mM sodium citrate solution (pH 8.0) at room temperature with gentle shaking for 0.5–1 min until complete dissolution. The released cells were immediately centrifuged at 6,000 rpm for 10 min, and the supernatant was discarded; the cell pellet was washed once with fresh A + medium to remove residual sodium citrate. Unencapsulated control cells were collected by centrifugation and subjected to the same washing procedure to ensure consistency. Cell density was measured as OD_730_ using a spectrophotometer. Chlorophyll fluorescence (F_v_/F_m_) was measured using a chlorophyll fluorometer after 15 min dark adaptation (Supplementary Method 1) (Sun et al. 2023). Photosynthetic oxygen evolution and dark respiration rates were determined using a YZQ-201A photosynthesis analyzer (Yizongqi Technology, China) (Supplementary Method 2) (Zhang et al. 2023). Cell viability was assessed by observing chlorophyll autofluorescence under a fluorescence microscope (Supplementary Method 3) (Xu, et al. 2025). Chlorophyll a and carotenoid contents were extracted in methanol, and quantified spectrophotometrically (Supplementary Method 4).

## Results

### Microbial contamination and phenotypic observations in large-scale cultivation of EP

**Fig. 1:**
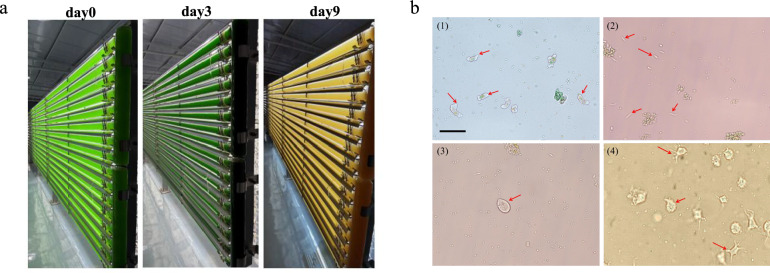
Culture failure and microbial contamination in scaled-up systems. **a** Macroscopic views of EP cultures grown in a 600 L glass tubular photobioreactor, captured on the inoculation day (left), day 3 (middle), and day 6 (right). The culture color transitions from pale green (initial inoculation) to dark green (transient growth) and finally to yellow–brown (culture collapse). **b** Representative micrographs of the complex contaminant community coexisting with the engineered cyanobacterium EP (protozoan predators marked with red arrows). The panel compiles typical fields of view from days 6–9 of cultivation, with minor background variations from differential sampling, and all images exhibit prominent contamination features. Scale bar, 20 μm

During attempts to scale up the cultivation of the ethanol-producing strain EP, we consistently observed severe culture failure across photobioreactors of various configurations, including a 100 L plate type, a 600 L glass-tubular type, and a 1000 L deep-tank type with internal illumination (Supplementary Figs. 1, 2). A representative example from a 600 L glass-tubular reactor shows that after inoculation, the culture initially turned dark green due to transient growth but faded to pale yellow or brown within days, signaling a complete culture collapse (Fig. 1a). This phenotypic deterioration coincided with a complete cessation of growth and undetectable ethanol production (Supplementary Fig. 3), reflecting a fundamental disruption of photosynthetic and metabolic activity.

Daily microscopic examination revealed that contaminating microorganisms were barely detectable on days 1–2, became visible on day 3 with the emergence of amoeboid protozoa, and were present in large numbers by day 6, forming a complex consortium that coexisted with EP cells (Fig. 1b, Supplementary Fig. 4). The contaminant consortium was diverse, consisting of multiple bacterial morphotypes that likely compete for essential nutrients and secrete inhibitory metabolites, as well as motile protozoa (e.g., flagellates and amoebae) that directly prey on cyanobacterial cells via phagocytosis, leading to rapid biomass loss (Nguyen et al. 2022). To characterize the contaminant community at the taxonomic level, 16S and 18S rRNA gene amplicon sequencing was performed (Supplementary Figs. 5 and 6). Among bacterial sequences, the engineered strain *Synechococcus* sp. PCC 7002 accounted for 39.6% (representing the background host). The most abundant contaminating bacterium was *Marinobacter* (19.1% of total bacterial sequences), followed by *Glycocaulis* (2.3%), *Halomonas* (1.0%), *Saccharospirillum* (1.0%), *Stappia* (0.7%), and other minor genera. The eukaryotic fraction was dominated by the algae *Plagiogrammopsis* (65.6%) and *Chloropicon* (26.5%), with only a trace of *Haematococcus* (0.1%). Together, these competing and predatory interactions highlight a key challenge for sustainable large-scale cultivation.

### Susceptibility of engineered strain EP to microbial contamination

**Fig. 2:**
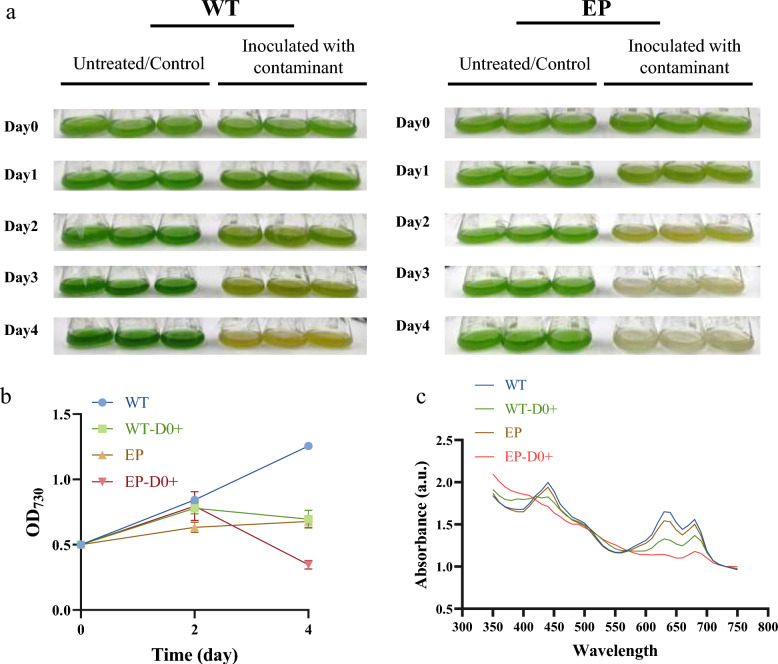
Phenotypic responses of wild-type (WT) and engineered (EP) strains to microbial contamination. **a** Visual comparison of uncontaminated controls and contaminated cultures over time. Cultures were inoculated at an initial OD_730_ of 0.5. Contaminated groups received the microbial consortium inoculum at a 1:10 (v/v) ratio, while controls received an equal volume of sterile medium. All cultures were incubated at 30 °C with continuous shaking at 100 rpm. Images are representative of three independent biological replicates. **b** Growth curves (OD_730_) of the four cultures. **c** Full-wavelength absorption spectra (350–750 nm) at day 2 post-inoculation. Groups: WT, control without contamination; WT‑D0 + , WT with contamination added at day 0; EP, control without contamination; EP‑D0 + , EP with contamination added at day 0. All experiments were performed with at least three independent biological replicates (n ≥ 3)

To evaluate and compare the susceptibility of the wild-type (WT) and the engineered ethanol-producing (EP) strains to the isolated contaminant consortium, contamination experiments were conducted under controlled conditions. A marked difference was observed between the two strains in their response to contamination pressure (Fig. 2a, b). When challenged with an identical contaminant inoculum, WT showed delayed collapse (complete by day 4), whereas strain EP suffered rapid and severe culture collapse within 48 h post-contamination, with cultures turning from deep green to pale yellow–brown, indicating massive cell lysis and culture failure.

Full-wavelength absorption spectra of the cultures at day 2 (Fig. 2c) revealed that EP under contamination stress exhibited marked reductions at wavelengths corresponding to key photosynthetic pigments: chlorophyll a (430 nm and 662 nm), carotenoids (446 nm and 474 nm), and phycobilisomes (625 nm), indicating severe pigment degradation and cell lysis (Rakhimberdieva et al. 2004; Méléder et al. 2013). In contrast, contaminated WT cultures retained higher pigment levels than contaminated EP cultures, although reduced relative to uncontaminated WT, consistent with their higher resistance compared to EP.

EP exhibited an inherently slower growth rate than WT and underwent more rapid cell death upon contamination, collectively suggesting its higher vulnerability. This disparity may be attributable to the metabolic burden imposed by the heterologous ethanol synthesis pathway (*pdc-adh*), which could reduce the competitive fitness of EP, making it more susceptible to microbial competition and predation, although this remains speculative and requires further comparative physiological and metabolomic analyses to test this possibility.

### Optimization of alginate concentration and characterization of bead stability

**Fig. 3:**
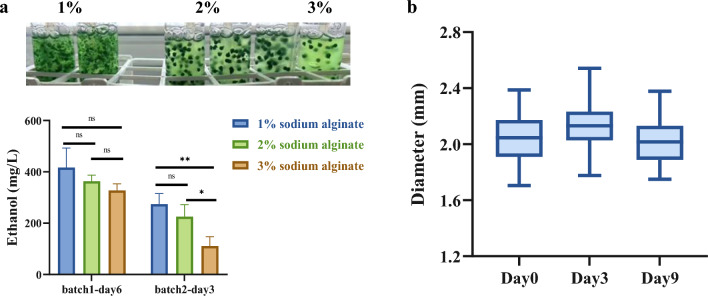
Optimization of alginate concentration and characterization of bead size distribution. **a** Effect of alginate concentration (1%, 2%, 3% w/v) on bead integrity and ethanol production. Experiments were conducted in 1 × A + medium. **b** Size distribution of alginate beads encapsulating cyanobacterial strain EP (n = 100). Bead diameters were measured using ImageJ software, and the distribution is shown as a box plot. All experiments were performed with at least three independent biological replicates (n ≥ 3), with data shown as mean ± SD. Statistical significance was determined by Student’s t‑test; *p < 0.05, **p < 0.01, ***p < 0.001; ns, not significant

Given the high susceptibility of engineered EP strain to microbial contamination, we next evaluated alginate encapsulation as a protective physical barrier strategy. To establish optimal conditions, we tested 1%, 2%, and 3% (w/v) alginate for encapsulation using two independent batch experiments (Fig. 3a). The first batch (6 days) evaluated initial bead integrity and ethanol yield. A second, extended batch (3 additional days under identical conditions) assessed long-term stability and mass-transfer performance. Beads made with 1% alginate disintegrated and leaked cells in both batches. The high ethanol yield observed was primarily due to the growth of these leaked cells in the medium, indicating a failure of the beads to provide effective encapsulation. While 3% alginate beads maintained stability and supported ethanol production in the first batch, yield markedly declined in the extended batch, suggesting that the denser gel hindered long-term nutrient and metabolite diffusion. In contrast, 2% alginate beads exhibited good structural integrity with only minor cell leakage, and supported stable ethanol production throughout both batches. This leakage may result from incomplete initial entrapment, mechanical stress due to aeration and agitation, or internal pressure from the growth and division of encapsulated cells. Such negligible leakage does not compromise overall bead stability or the validity of the encapsulation strategy. Consequently, 2% alginate was selected for all further studies.

Using 2% alginate, the resulting beads exhibited a uniform size distribution and remained stable in diameter throughout the cultivation period (Fig. 3b). Nitrate concentrations in the medium were significantly lower at days 3 and 6 than at day 0 (Supplementary Fig. 7, Supplementary Method 5), confirming nutrient diffusion through the alginate beads. Under aerated culture conditions, the beads retained their integrity throughout the 15‑day cultivation period (196–198 intact beads out of 200 initial beads; Supplementary Fig. 8).

### Encapsulation effect on cell performance and ethanol yield

**Fig. 4:**
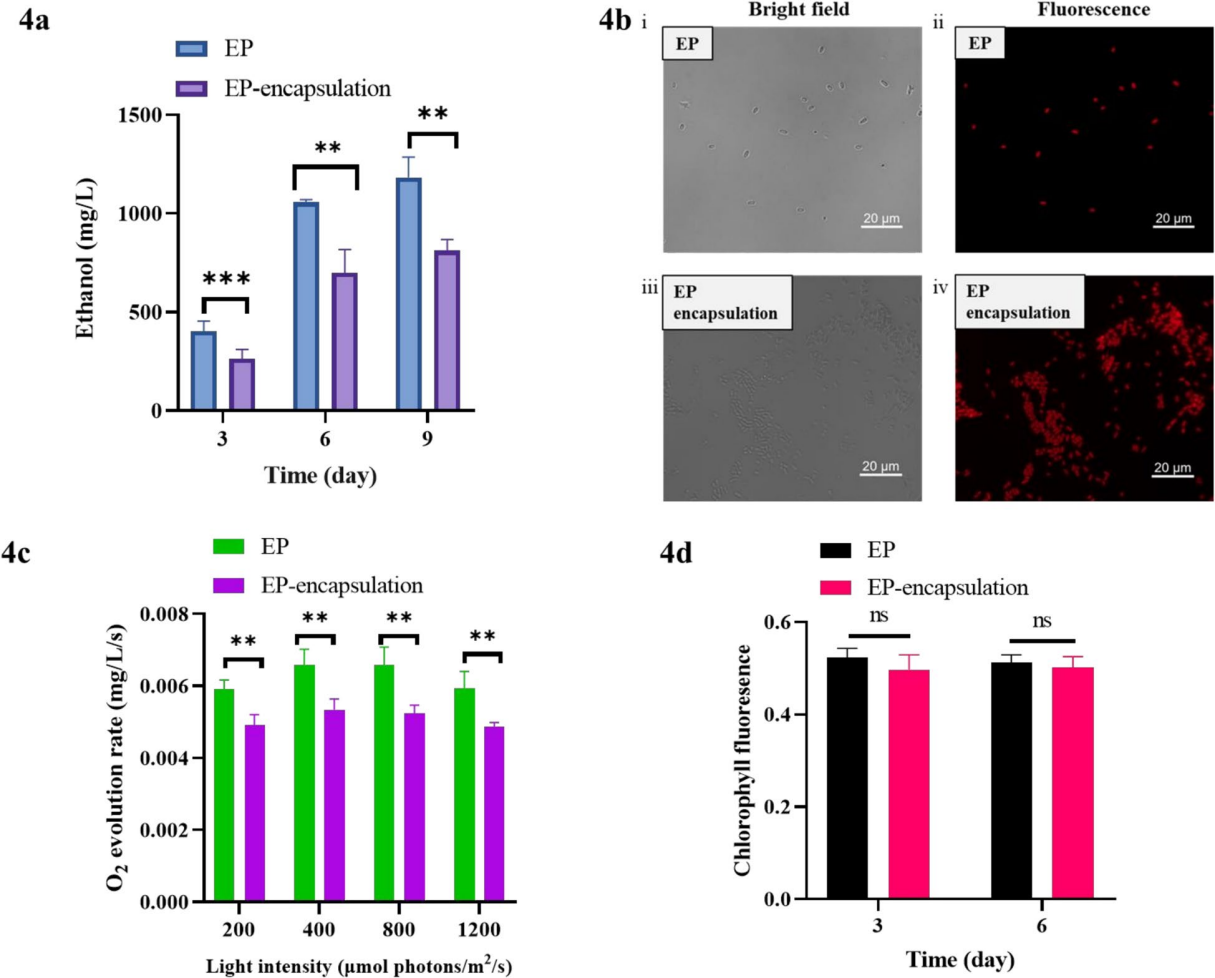
Performance of unencapsulated and alginate-encapsulated EP cells under axenic conditions. **a** Ethanol production of unencapsulated EP cells and alginate-encapsulated EP cells under axenic conditions. **b** Representative bright-field and Cy5 fluorescence images of EP cells under uncontaminated conditions on day 6. (i) Bright-field of unencapsulated EP cells; (ii) Cy5 fluorescence of unencapsulated cells; (iii) bright-field of alginate beads; (iv) corresponding Cy5 fluorescence image showing chlorophyll autofluorescence of encapsulated cells. Scale bars = 20 μm. **c** Photosynthetic oxygen evolution rates of unencapsulated and encapsulated EP cells on day 6. **d** Maximum quantum efficiency of photosystem II (F_v_/F_m_) of unencapsulated and encapsulated EP cells on day 3 and 6. All experiments were performed with at least three independent biological replicates (n ≥ 3), with data shown as mean ± SD. Statistical significance was determined by Student’s t‑test; *p < 0.05, **p < 0.01, ***p < 0.001; ns, not significant

Using the optimized 2% alginate condition established above, we next evaluated how encapsulation influences cell performance and ethanol production under contaminant‑free conditions. In a comparative axenic study, we established two groups: encapsulated (2% alginate) and unencapsulated (control) cultures of strain EP. Ethanol production was confirmed in both systems, indicating that encapsulation did not inactivate the ethanologenic pathway in EP. However, the encapsulated group showed a significantly lower cumulative ethanol yield than the control over time (Fig. 4a). At day 9, the cumulative yield of encapsulated cultures was 700 mg/L, compared to 1056 mg/L for unencapsulated controls.

To understand the physiological basis for this yield reduction, we assessed the viability and photosynthetic activity of encapsulated cells on day 6. Fluorescence microscopy of bead cross-sections revealed abundant viable cells exhibiting red chlorophyll autofluorescence throughout the beads (Fig. 4b). Beads were then dissolved using sodium citrate to release the entrapped cells. Photosynthetic oxygen evolution rates showed that encapsulated cells maintained high net photosynthetic activity, although the rates were slightly lower than those of unencapsulated cells (Fig. 4c). Chlorophyll fluorescence measurements showed that the maximum quantum efficiency of photosystem II (F_v_/F_m_) of released cells was not significantly different from that of unencapsulated cells (Fig. 4d). Chlorophyll a content and full‑wavelength absorption spectra showed no significant differences between encapsulated and unencapsulated cells, whereas carotenoid contents were lower in the encapsulated group (Supplementary Fig. 9). In terms of final biomass, the released encapsulated cells reached an OD_730_ of 7.4, compared to 11.8 for unencapsulated control cultures (Supplementary Fig. 9).

The unchanged F_v_/F_m_ values, together with the unaltered chlorophyll a content and absorption spectra, indicate that encapsulation did not impair the structural or functional integrity of the photosynthetic apparatus. The moderate reduction in net photosynthetic oxygen evolution, and consequently in biomass accumulation and ethanol yield, therefore likely reflects physical constraints on substrate availability rather than damage to the photosynthetic machinery. As cells proliferated within the beads, mutual shading may have created light gradients that limited the effective photon flux reaching interior cells, while the alginate hydrogel matrix could potentially impose diffusion limitations on inorganic carbon and nutrients. These combined physical constraints represent a possible explanation for the lower productivity observed. Future studies examining intra-bead light distribution and solute diffusion are needed to elucidate the relative contributions of these physical factors.

### Protection conferred by alginate encapsulation against microbial contamination

**Fig. 5:**
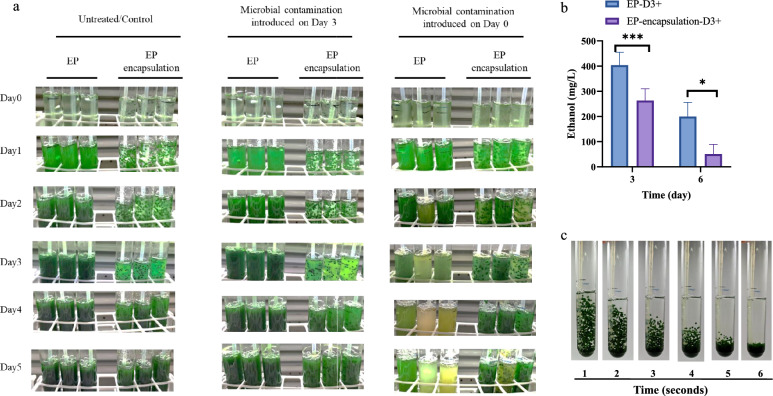
Effect of encapsulation and contamination timing on culture phenotype and ethanol production. **a** Culture phenotypes under different experimental conditions. The panels compare unencapsulated (left) and alginate-encapsulated (right) cultures of strain EP across three contamination regimes: no contamination (control), contamination introduced on day 3, and contamination introduced on day 0. **b** Ethanol production profiles corresponding to the condition of contaminant addition on day 3. EP, unencapsulated control; EP-encapsulation, alginate-encapsulated without contamination; EP-D3 + , unencapsulated with contamination on day 3; EP-encapsulation-D3 + , encapsulated with contamination on day 3. All experiments were performed with at least three independent biological replicates (n ≥ 3), with data shown as mean ± SD. Statistical significance was determined by Student’s t‑test; *p < 0.05, **p < 0.01, ***p < 0.001; ns, not significant. **c** Sedimentation of alginate microbeads under static conditions, demonstrating rapid biomass recovery. Representative photographs taken at 1, 2, 3, 4, 5, and 6 s after placement under static conditions, showing the rapid settling of beads

To evaluate the impact of complex microbial contamination on ethanol synthesis and the protective efficacy of alginate encapsulation, the contaminant consortium was introduced to cultures of strain EP at two time points: day 0 and day 3. Ethanol production and culture phenotypes were then compared between unencapsulated and encapsulated cultures (Fig. 5a, b). Encapsulation provided striking protection against early contamination (day 0). While unencapsulated cultures collapsed rapidly, alginate-encapsulated EP cells maintained viability and growth under contamination pressure. These results suggest that alginate encapsulation provides a physical barrier that prevents culture collapse and preserves metabolic function.

When contamination was delayed until day 3, both systems initially remained viable, suggesting that established biomass conferred temporary resilience. However, upon contaminant addition, ethanol production ceased abruptly, with cumulative yields dropping from > 400 mg/L to undetectable levels (Fig. 5b). Since ethanol is secreted into the medium, it becomes accessible to heterotrophic contaminants. To determine whether the observed drop in cumulative ethanol resulted from consumption rather than cessation of production, we incubated the isolated contaminant consortium in medium containing 1 g/L ethanol (experimental conditions: 30 °C, 100 rpm, in A + medium); within 24 h, ethanol was completely depleted. Moreover, the contaminating organisms may also consume nutrients present in the culture medium, creating additional metabolic competition with the encapsulated cyanobacteria. Thus, while encapsulation effectively prevents culture collapse, it does not shield the extracellular medium from contaminant activity, and this limitation must be considered for extracellular products such as ethanol.

Beyond its protective role, encapsulation may confer a practical benefit for downstream processing. To evaluate harvesting efficiency, we compared the sedimentation of encapsulated EP cells with that of unencapsulated cell suspensions. Under static conditions, alginate beads settled completely within 10 s, enabling quantitative biomass recovery (Fig. 5c). This rapid sedimentation simplifies biomass harvesting under static laboratory conditions and may represent a potential advantage for reducing energy consumption in downstream processing compared to centrifugation.

### Recovery of ethanol production in rinsed alginate-encapsulated EP

Despite the limitations of a physical barrier against metabolic competition and product consumption, alginate encapsulation successfully prevented culture collapse and preserved cell viability under contamination pressure. To determine whether the shielded biomass could regain metabolic function after contaminants were removed, recovery experiments were performed under two regimes: mid-cultivation (Condition 1, Supplementary Fig. 10) and early-stage (Condition 2) contamination. For recovery, cultures were harvested 2 days post-contamination in Condition 1 (day 2 contamination). Alginate beads were collected by sedimentation, and unencapsulated cells by centrifugation. All samples were then washed and transferred to fresh medium. In Condition 2 (day 0 contamination), only encapsulated beads were processed for recovery after 3 days, as unencapsulated cultures had completely failed.

**Fig. 6:**
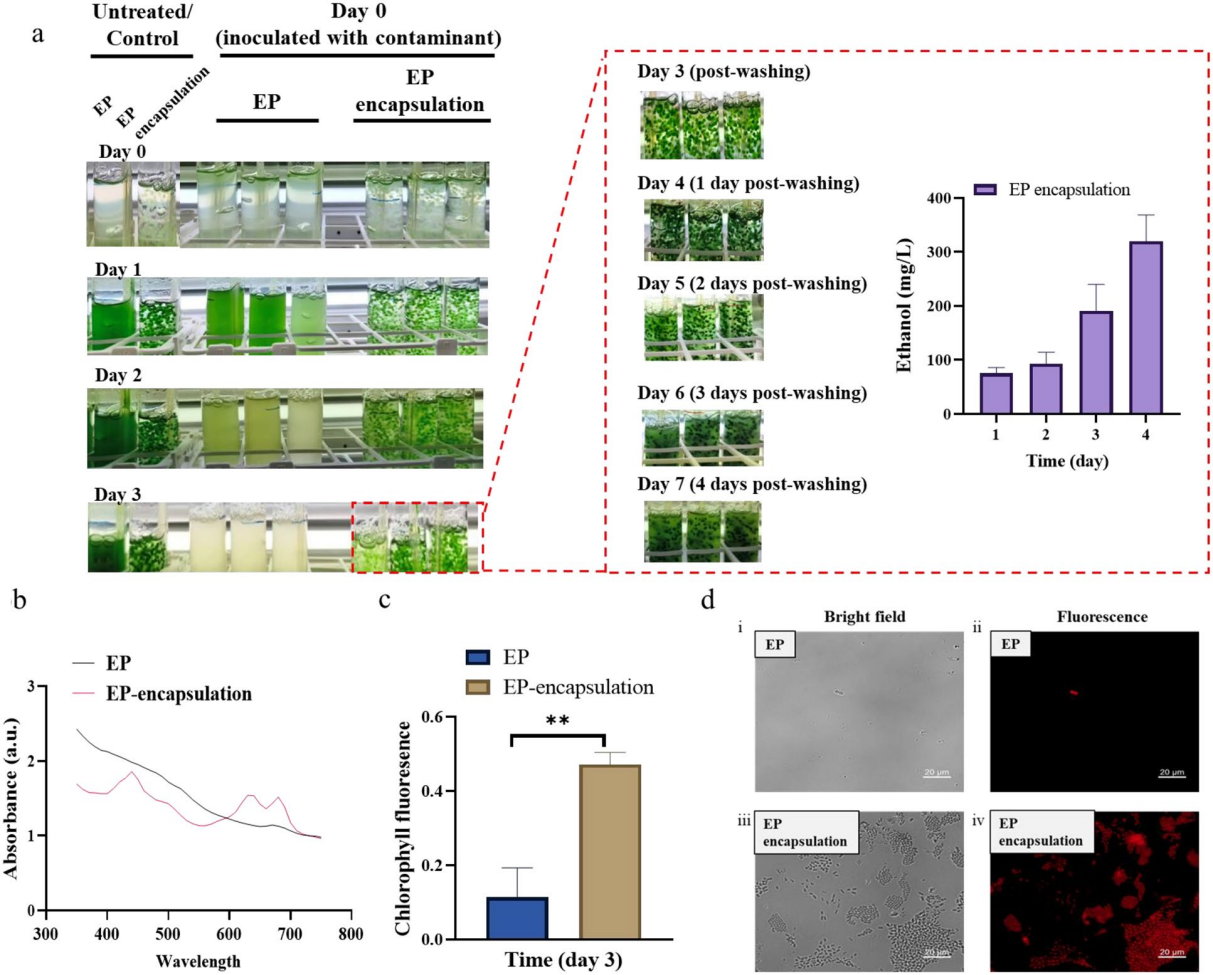
Functional recovery of alginate-encapsulated EP following contamination and washing. **a** Recovery of encapsulated cells under early‑stage contamination (Condition 2). Contamination was introduced at day 0. After 3 days, only the alginate-encapsulated beads survived, whereas unencapsulated cultures had completely collapsed. A red border highlights the recovery phase (post-washing), showing the culture image and the ethanol production profile over the subsequent 4 days. **b** Full-wavelength absorption spectra (350–750 nm) of unencapsulated and encapsulated cells after 3 days under contamination (Condition 2). **c** Chlorophyll fluorescence parameter F_v_/F_m_, indicating the maximum quantum efficiency of photosystem II. **d** Fluorescence microscopy of unencapsulated and encapsulated cells under contamination pressure (Condition 2, day 3). Unencapsulated cells showed only a few scattered surviving cells with strongly reduced red chlorophyll autofluorescence, whereas encapsulated beads exhibited abundant viable cells with bright autofluorescence throughout the beads. Scale bar = 20 μm. All experiments were performed with at least three independent biological replicates (n ≥ 3), with data shown as mean ± SD. Statistical significance was determined by Student’s t‑test; *p < 0.05, **p < 0.01, ***p < 0.001; ns, not significant

Recovery outcomes indicated the efficacy of encapsulation. In Condition 1, washed unencapsulated cultures exhibited no recovery and were non-viable by day 4 post-transfer. In contrast, washed alginate beads retained viability and restored partial ethanol productivity, yielding a cumulative ethanol production of ~ 180 mg/L (Supplementary Fig. 10). Under the more severe early-contamination scenario (Condition 2), the washed encapsulated beads not only survived but also regained considerable metabolic activity, achieving a cumulative ethanol production of ~ 320 mg/L (Fig. 6a).

To characterize the physiological state of the cultures in the early‑contamination scenario (Condition 2), key photosynthetic parameters were assessed at day 3 post‑contamination. In unencapsulated cultures, full‑wavelength absorption spectra were barely detectable (Fig. 6b), and F_v_/F_m_ as well as photosynthetic oxygen evolution showed no measurable increase over baseline (Fig. 6c, Supplementary Fig. 11); fluorescence microscopy revealed only a few scattered surviving cells with significantly reduced autofluorescence (Fig. 6d). In contrast, encapsulated cultures retained fully normal values for all these parameters, and fluorescence microscopy showed abundant viable cells exhibiting bright red chlorophyll autofluorescence throughout the beads. Genotypic analysis performed at the end of the cultivation period suggested that both engineered loci remained fully integrated in the recovered cells, indicating genetic stability throughout the contamination and recovery process (Supplementary Fig. 12).

These results suggest a dual advantage of alginate encapsulation: it provides an effective physical barrier that sustains culture viability during contamination episodes, and facilitates functional recovery of the production system through simple washing and re-inoculation. This operational resilience may represent a promising feature for future development of cyanobacterial cultivation processes.

## Discussion

This study suggests that alginate encapsulation could potentially contribute to protecting engineered, ethanol-producing cyanobacteria from microbial contamination, a major obstacle to scalable cultivation. We first confirmed that the engineered EP strain is significantly more susceptible to a complex environmental contaminant consortium than its wild-type parent. Given this susceptibility, we examined alginate encapsulation as a potential physical barrier for protecting EP cells. This approach functions by forming a size-exclusion barrier that helps reduce direct contact and ingestion by contaminants, yet remains permeable to gases, light, and nutrients, thus helping maintain core photosynthetic and metabolic functions. Critically, encapsulated cultures not only resisted contamination but also regained ethanol productivity after a simple rinsing and re-inoculation step. This indicates that physical containment can enable functional recovery of a production system after contamination.

However, the current encapsulation system may have notable limitations in internal mass transfer and light availability. Cell proliferation within the gel creates a high internal density, leading to self-shading by outer-layer cells. This is likely to create a steep light gradient within the bead core, which could reduce overall photosynthetic efficiency and constrain metabolic output. Future work should address these limitations by measuring the effective diffusion coefficients of key solutes (e.g., CO_2_, bicarbonate, ethanol) within the alginate matrix using diffusion cells or microelectrodes (Cherenkov et al. 2024; Nalzaro and Tumolva 2025). Such insights would enable rational design strategies, including (i) controlling the initial cell loading to avoid overcrowding, (ii) optimizing bead size and gel porosity to enhance diffusion, (iii) developing composite matrices with improved light-transmitting properties, and (iv) enhancing mechanical stability through composite materials or surface coatings to extend functional integrity for long-term cultivation.

Beyond controlling biocontamination, this encapsulation strategy may offer operational and economic benefits for cultivation systems. Alginate beads enable rapid static sedimentation at the laboratory scale, which could simplify biomass harvesting relative to centrifugation-based recovery. However, the practical benefits at industrial scale will depend on bead stability under continuous operation, harvesting efficiency in large volumes, and the overall economics of bead production and reuse. While the material and preparation costs of encapsulation are non-negligible, these may be offset by reduced contamination-induced batch failures and the elimination of cell-gel separation steps (leveraging ethanol secretion). A comprehensive techno-economic assessment under pilot-scale conditions is warranted to evaluate the viability of this approach for commodity ethanol production.

## Supplementary Information

Supplementary Material

## Data Availability

The datasets used and/or analysed during the current study are available from the corresponding author on reasonable request.

## Abbreviations

PDC: Pyruvate decarboxylase
ADH: Alcohol dehydrogenase
WT: Wild-type
EP: Ethanol-producing strain (1 + 2PA)
