# Supplementary material for: Mitigation of microbial biocontamination in cyanobacterial ethanol synthesis via alginate encapsulation
Source: Bioresour Bioprocess. 2026 May 26;13(1):77. doi: 10.1186/s40643-026-01076-7 (PMC13212877; doi:10.1186/s40643-026-01076-7)
Supplement: Supplementary file 1 — Supplementary Material [file 40643_2026_1076_MOESM1_ESM.docx]

**Supplementary Material**

**Supplementary Methods**

**Supplementary Method 1. Determination of Chlorophyll Fluorescence Parameter (**F_v_/F_m_**)**

Cells released from alginate beads by sodium citrate dissolution were resuspended in fresh A+ medium, adjusted to an OD_730_ of 0.8, and were dark-adapted at room temperature for at least 15 minutes. Chlorophyll fluorescence was measured using a Dual-PAM-100 pulse-amplitude modulated fluorometer (Heinz Walz, Germany). The instrument was operated in fluorescence mode with a DPD (Dual-PAM Detector) high-sensitivity detector. Prior to sample addition, the measuring light (ML) intensity was adjusted based on the initial fluorescence signal of the sample to maintain a stable baseline between 0.2 and 0.5. The actinic light (AL) intensity was set to half-saturation light intensity. Measurement was performed under dark conditions with ML only. After transferring the sample into a cuvette, the manual measurement mode was initiated by clicking “START”, followed by “F_0_, F_m_” to record the minimum fluorescence (F_0_). Subsequently, AL was turned on and DCMU was added to the sample. The sample was gently mixed, and the fluorescence signal was monitored until stabilization. During this period, the “Fluo sp” button was intermittently clicked to record the maximum fluorescence (F_m_). The F_v_/F_m_ value was calculated as (F_m_ − F_0_)/F_m_.

**Supplementary Method 2. Determination of Total Photosynthetic Oxygen Evolution Rate.** Cyanobacterial cells released by sodium citrate dissolution were resuspended in fresh A+ medium and adjusted to an OD_730_ of 1.0, with the detection temperature set at 30 °C. Real-time changes in oxygen concentration in the sample chamber were monitored using a YZQ-201A Photosynthetic Oxygen Evolution Analyzer (Yizongqi, China). 17 mL of the algal suspension was mixed with 10 mM NaHCO_3_ and injected into the sample chamber. The perforated conical cap was tightened to expel air, followed by hermetic sealing with a rubber stopper. The measurement program was set as follows: a dark stabilization period of 250 s, followed by sequential detection for 320 s under dark conditions and light intensities of 200, 400, 600, 800 and 1200 μmol photons/m^2^/s, respectively. The analyzer automatically recorded the oxygen concentration (mg/L, defined as the oxygen content in 1 L of algal suspension with an OD_730_ of 1.0). For data processing, the slope was calculated using the data from the last 100 s under each light condition to obtain the net photosynthetic oxygen evolution rate (mg/L/s). The total photosynthetic oxygen evolution rate was determined by adding the dark respiration rate to the net photosynthetic oxygen evolution rate.

**Supplementary Method 3. Assessment of Cell Viability by Fluorescence Microscopy.** To assess cell viability, encapsulated samples were first prepared by gently slicing alginate beads into thin sections using a sterile scalpel. A single bead section was placed on a glass slide, covered with a coverslip, and gently pressed to immobilize the sample. For unencapsulated samples, 5 μL of cell suspension was transferred directly onto a glass slide and covered with a coverslip. Bright-field and fluorescence observations were performed using a Zeiss Axio Imager Z2 fluorescence microscope equipped with a 40× objective lens. For fluorescence imaging, the Cy5 filter set was used to detect chlorophyll autofluorescence, and the exposure time was uniformly set to 10 ms. Viable cells exhibited red fluorescence, while non-viable or damaged cells showed significantly reduced or absent fluorescence intensity.

**Supplementary Method 4. Extraction and Determination of Chlorophyll a.** 1 mL of cyanobacterial suspension was collected and centrifuged, and the supernatant was discarded thoroughly. The algal cell pellet was resuspended in 1 mL of methanol, and gently disrupted with a pipette tip to ensure complete cell dispersion. The mixture was then incubated at -20℃ in the dark overnight to facilitate sufficient pigment extraction. After incubation, the sample was centrifuged at 13,000 rpm for 5 min, and the supernatant extract was collected for subsequent detection. The absorbance values at 665 nm and 720 nm were measured using a spectrophotometer, with methanol set as the blank control.

**Supplementary Method 5. Ion Chromatography Analysis of Inorganic Nutrients.** Inorganic ion concentrations in the culture medium were determined using a Dionex ICS-5000 ion chromatography system (Thermo Fisher Scientific, USA). Cations were separated on a CS12A column with 20 mM methanesulfonic acid as the mobile phase at a flow rate of 1.0 mL/min. Anions were separated on an AS11-HC column with 25 mM sodium hydroxide as the mobile phase at a flow rate of 1.0 mL/min. Prior to injection, all samples were filtered through a 0.22 μm membrane. The injection volume was 25 μL. Ion concentrations were quantified using external standard curves.

**Supplementary Figures**


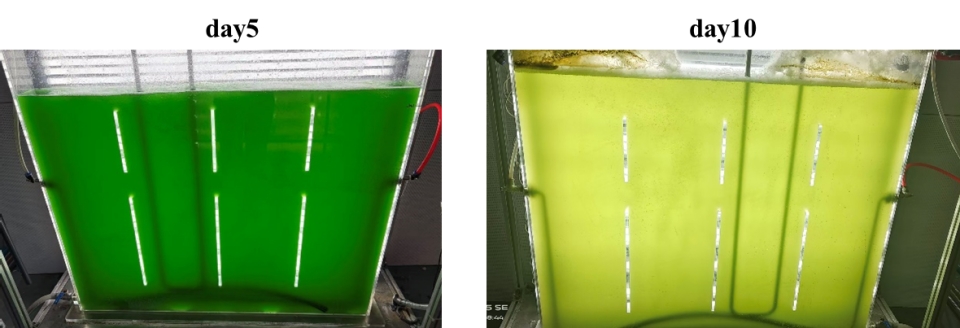


**Supplementary Figure 1. Visual documentation of culture collapse in a 100 L plate photobioreactor.** Prior to cultivation, the reactor was sterilized with sodium hypochlorite, thoroughly rinsed with purified water, and sealed with a gasket secured by bolts. The reactor was inoculated using a culture from indoor 10 L glass flasks at an initial optical density (OD_730_) of approximately 0.3-0.5. Both the inoculum and the growth medium (A+ medium supplemented with twofold nitrogen and phosphorus) were pumped into the reactor via a side valve to achieve a 100 L working volume. Illumination was provided by external fluorescent tubes at an intensity of about 150 μmol photons/m^2^/s. A mixed gas stream of air containing 3% CO_2_ was continuously supplied through nano-diffusers located at the bottom, serving as both the carbon source and the means of mixing.


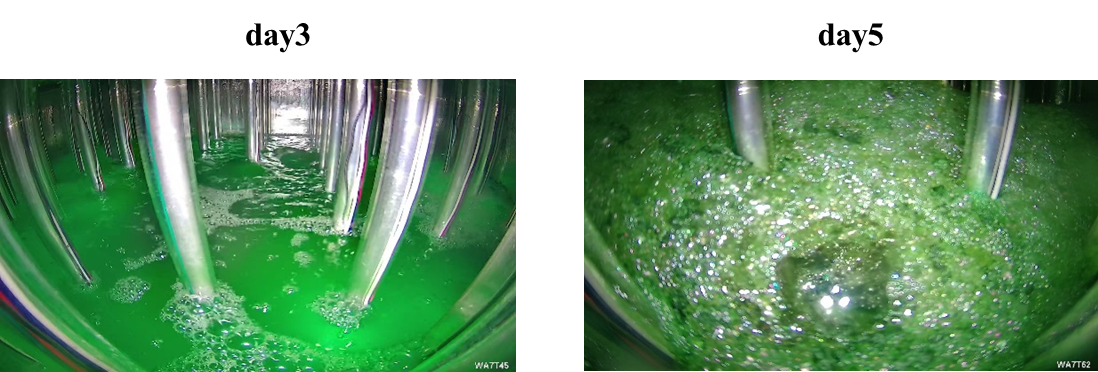


**Supplementary Figure 2. Scale-up cultivation of the ethanol-producing cyanobacterium in a 1000 L deep-tank photobioreactor equipped with internal illumination.** The reactor was sterilized with sodium hypochlorite and citric acid prior to use. Nutrients (A+ medium with twofold nitrogen and phosphorus) were dissolved in a mixing tank, filter-sterilized through a 0.22 μm membrane, and transferred into the reactor. The culture was initiated by aseptically pumping in 100 L of a laboratory-grown inoculum to achieve a final working volume of 1000 L. Cultivation conditions were maintained with internal LED lighting (adjusted based on cell density), bottom agitation/aeration, pH control (7.5–7.8 via automated CO_2_ supply), and temperature regulation (28–32 °C via a cooling circulation system). Although initial growth was observed after inoculation, the culture collapsed by day 4, exhibiting extensive foaming due to microbial metabolic activity and contamination was confirmed by microscopy.

**
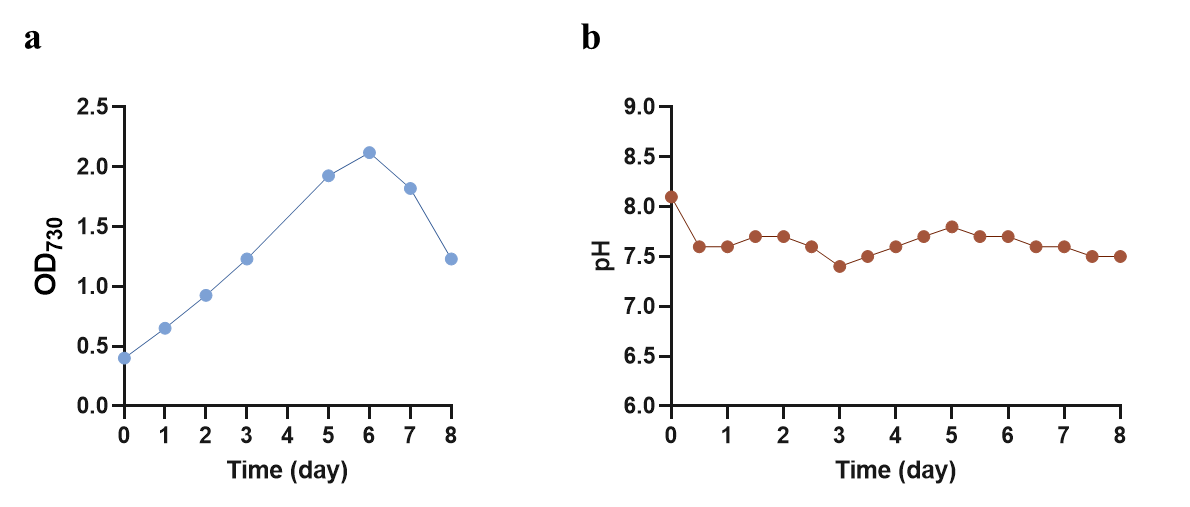
**

**Supplementary Figure 3. Growth profiles (a) and pH (b) of the EP culture grown in a 600 L glass tubular photobioreactor.** Cultivation was performed in 1× A+ medium with twofold concentrations of nitrogen and phosphorus (2× N, 2× P), under continuous illumination at a photon flux density of approximately 100 μmol photons/m^2^/s. The temperature was maintained between 28 and 32 °C using a cooling‑jacket system controlled by a chiller (activated above 32 °C, deactivated below 28 °C). The pH was automatically regulated between 7.5 and 7.8 by solenoid‑valve‑controlled CO_2_ supplementation (CO_2_ on when pH > 7.8; off when pH < 7.5), while air was supplied continuously. Prior to inoculation, all tubing and reactor surfaces were sterilized with sodium hypochlorite, and all liquids used were filtered through 0.22 μm membranes.


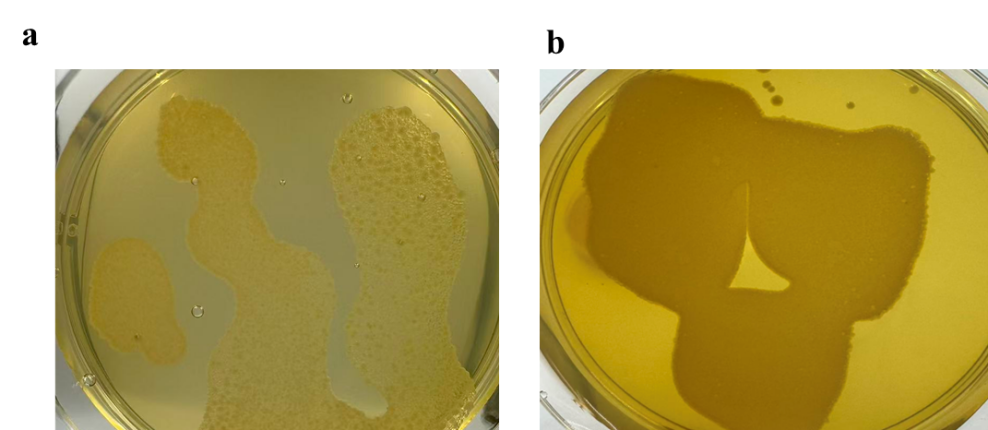


**Supplementary Figure 4. Representative LB agar plate inoculated with a sample from a collapsed EP culture.** After incubation at 37°C for 24 hours, dense and morphologically diverse bacterial colonies were observed, directly confirming severe heterotrophic contamination.


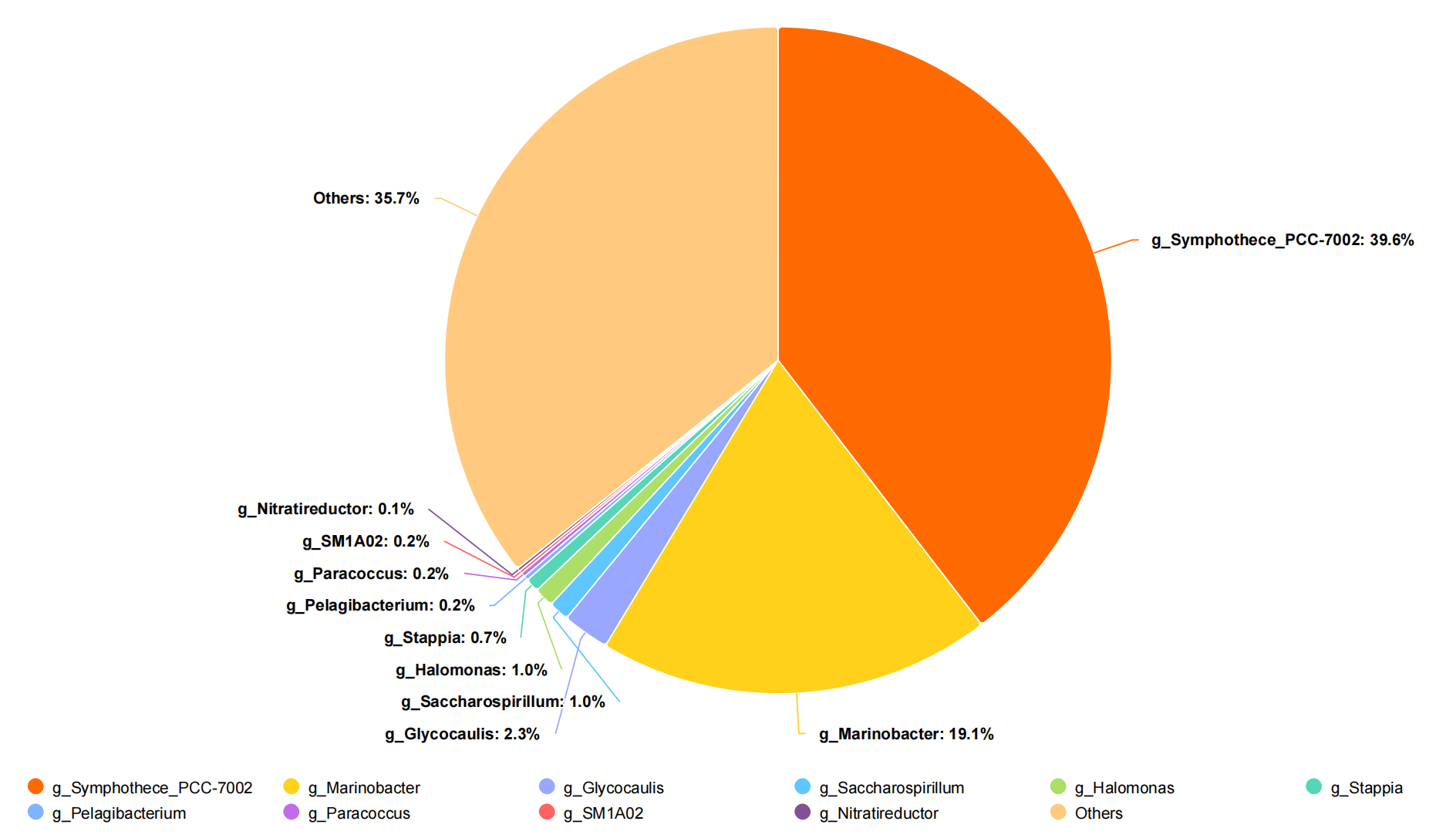


**Supplementary Figure 5. Bacterial community structure of the contaminant consortium (16S rRNA amplicon sequencing).** Cell pellets collected from the contaminant consortium were sent directly to Personal Biotechnology Co., Ltd. (Shanghai, China) for 16S rRNA gene sequencing. The V3–V4 region was amplified using primers 338F and 806R. Taxonomy was assigned using the SILVA database. The pie chart shows the relative abundance of bacterial genera. The engineered host strain, *Synechococcus* sp. PCC 7002, was annotated in the sequencing database as *Symphothece* PCC 7002 (a synonymous or database-specific designation). It accounted for 39.6% of the total bacterial sequences. Among the true contaminants, *Marinobacter* was the most abundant (19.1%), followed by *Glycocaulis* (2.3%), *Halomonas* (1.0%), *Saccharospirillum* (1.0%), and *Stappia* (0.7%). The taxon labelled “g_SM1A02” (0.2%) represents an unclassified or uncultured bacterial group commonly detected in environmental samples; its exact taxonomic position remains unresolved. Other genera with relative abundances below 0.5% were pooled as “Others”.


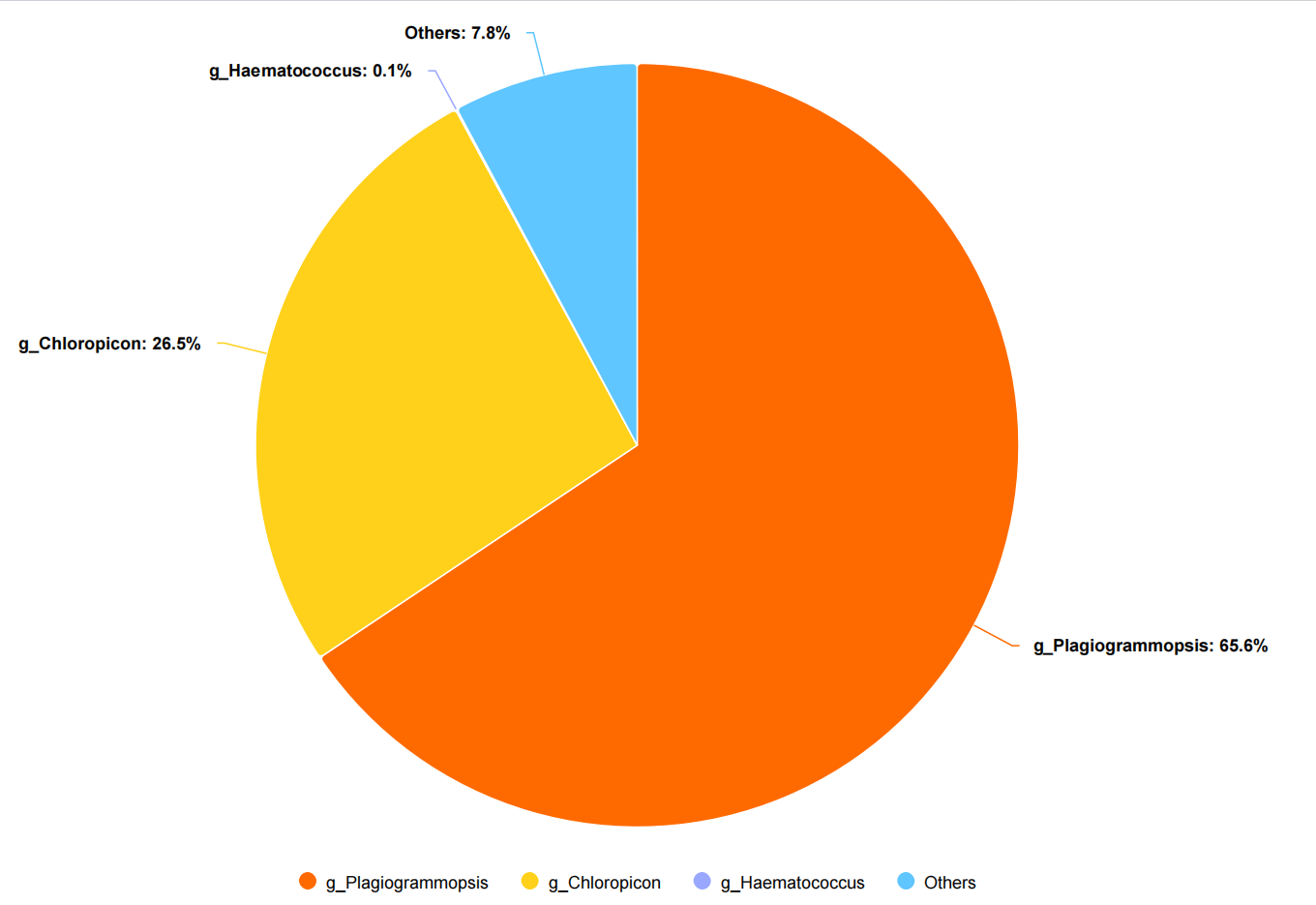


**Supplementary Figure 6. Eukaryotic community composition of the contaminant consortium (18S rRNA amplicon sequencing).** The same cell pellets were used for 18S rRNA gene sequencing. Personal Biotechnology Co., Ltd. (Shanghai, China) performed DNA extraction and sequencing. The V4 region was amplified with primers 547F and V4R. Taxonomy was assigned using the SILVA database. The eukaryotic fraction was overwhelmingly dominated by two algal genera: *Plagiogrammopsis* (65.6%) and *Chloropicon* (26.5%). *Haematococcus* represented only 0.1% of the sequences. The remaining 7.8% comprised other minor or unclassified eukaryotes.

**Supplementary Figure 7. Nitrate consumption in the culture medium during cultivation of encapsulated and unencapsulated cyanobacteria.** Nitrate concentrations (mg/L) were measured at days 0, 3, and 6. Data are presented as mean ± SD (n≥3).


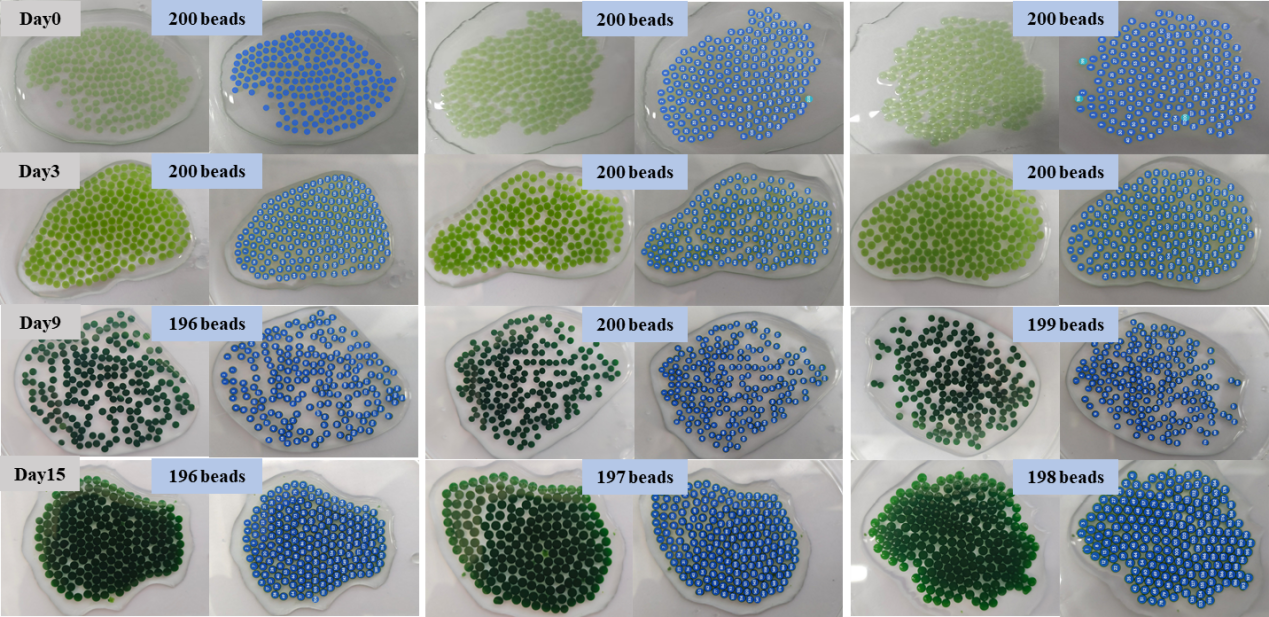


**Supplementary Figure 8. Long-term integrity of alginate beads under aerated conditions.** Representative images of alginate beads captured at days 0, 3, 9, and 15 of cultivation. Over the 15-day period, the beads maintained their structural integrity, with only minimal loss (2–4 beads per replicate).


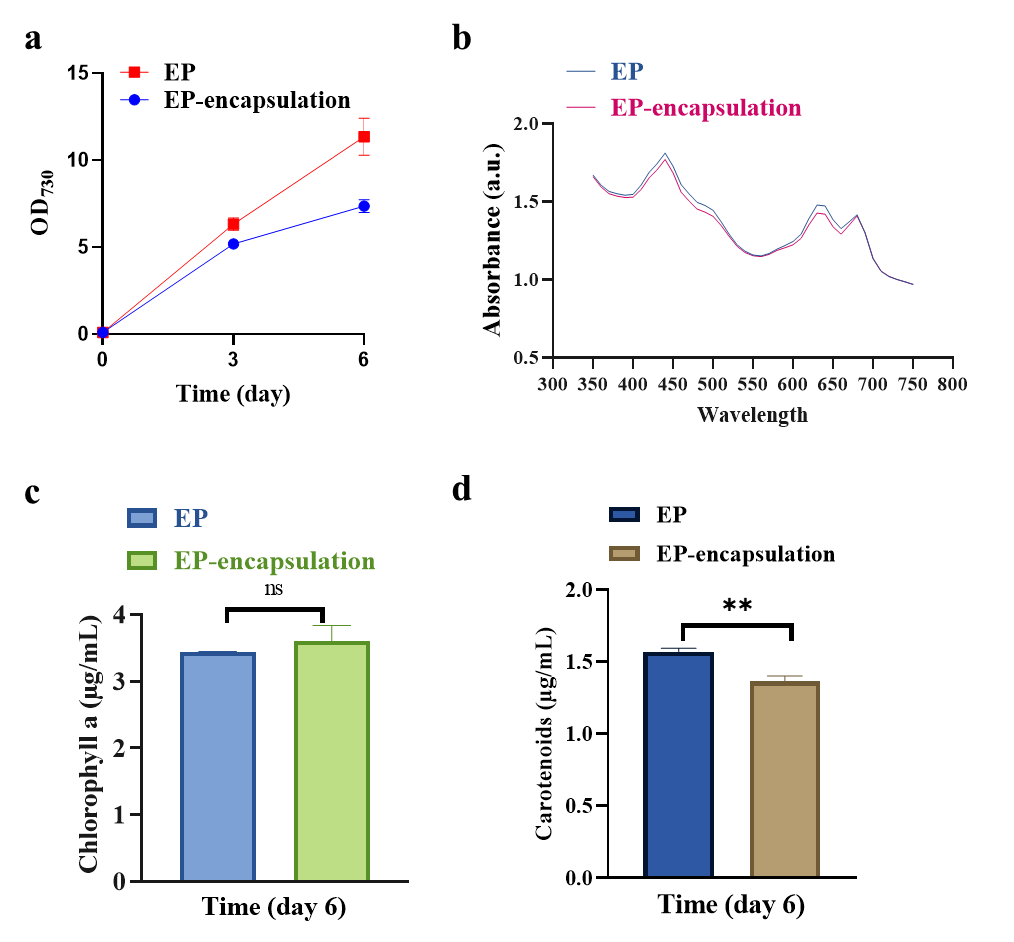


**Supplementary Figure 9. Growth and pigment characterization of encapsulated and unencapsulated EP cells.** For encapsulated samples, cells were first released from alginate beads by sodium citrate dissolution prior to all measurements. Free cell samples were processed in parallel using the same procedure for consistency. (a) Optical density at 730 nm (OD_730_) of unencapsulated and encapsulated cultures, with values for encapsulated cells obtained after bead dissolution and normalized to the original culture volume, compared at the same time points. (b) Full-wavelength absorption spectra (350-750 nm) of encapsulated and free cells. (c) Chlorophyll a content (mg/L). (d) Carotenoid content (mg/L). Data are presented as mean±SD (n≥3). Statistical significance was determined by unpaired two-tailed Student’s t-test.


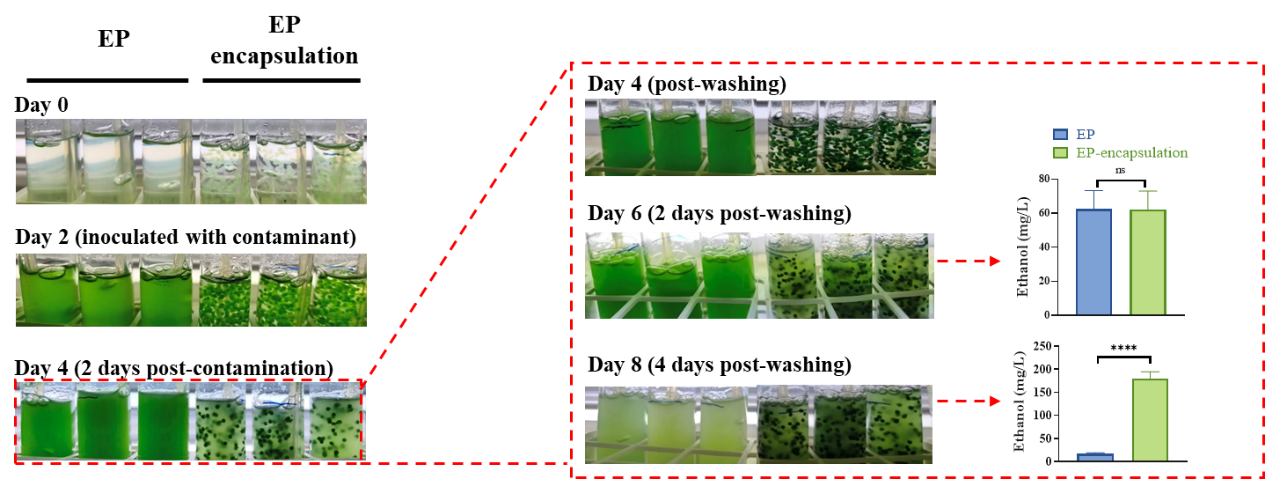


**Supplementary Figure 10.** Functional recovery of encapsulated EP under mid‑cultivation contamination (Condition 1). Contamination was introduced on day 2. Culture status before (days 0–2) and after (days 2–4) contamination is shown. The recovery phase (post‑washing and re‑inoculation), highlighted with a red border, includes the culture image and ethanol production profile at 2 and 4 days post‑recovery.


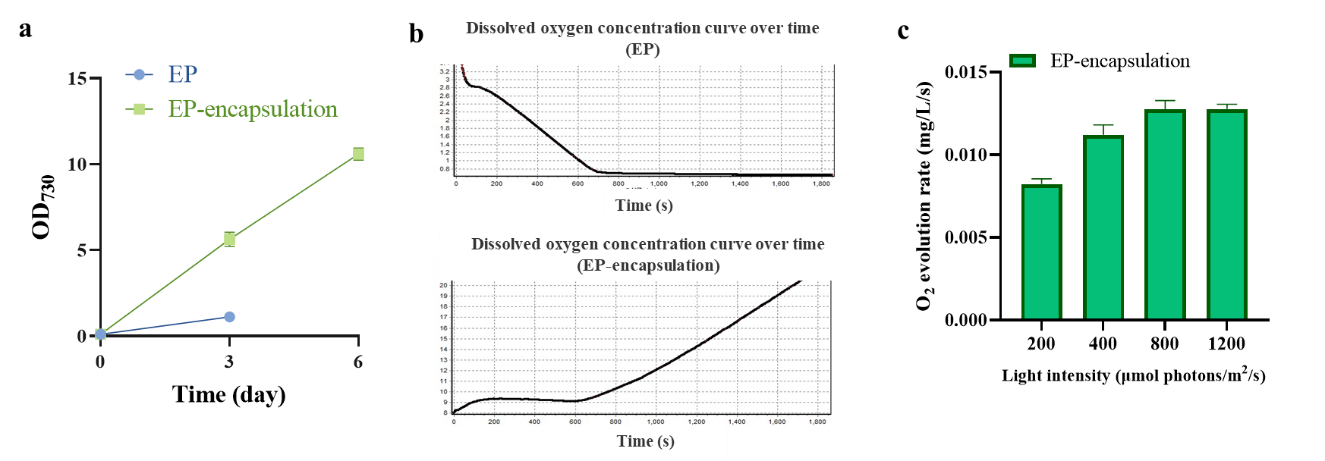


**Supplementary Figure 11. Photosynthetic characteristics of unencapsulated and encapsulated EP cells after contamination challenge (Condition 2).** Cells were released from alginate beads by sodium citrate dissolution prior to all measurements. a) Optical density at 730 nm (OD_730_) of unencapsulated and encapsulated cultures. b) Representative oxygen evolution traces recorded in the oxygen electrode chamber. Upper trace: unencapsulated control (no detectable activity); lower trace: encapsulated cells (maintained oxygen evolution). c) Whole-chain photosynthetic oxygen evolution rates of the encapsulated cells (quantitative data). Data are presented as mean ± SD (n≥3).


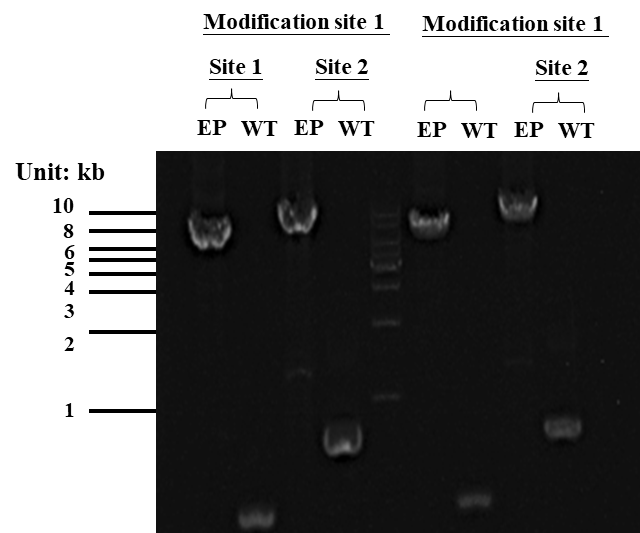


**Supplementary Figure 12. PCR verification of the integrated *pdc-adh* cassette in strain EP before and after cultivation.** PCR amplification of genomic DNA extracted before and after cultivation confirmed the stable integration of the *pdc-adh* cassette, as evidenced by the expected band size and 100% sequence identity.
